# Natural Compounds from Saffron and Bear Bile Prevent Vision Loss and Retinal Degeneration

**DOI:** 10.3390/molecules200813875

**Published:** 2015-07-31

**Authors:** Laura Fernández-Sánchez, Pedro Lax, Agustina Noailles, Antonia Angulo, Victoria Maneu, Nicolás Cuenca

**Affiliations:** 1 Departament of Physiology, Genetics and Microbiology, University of Alicante, 03690 Alicante, Spain; E-Mails: laura.fs@ua.es (L.F.-S.); pedro.lax@ua.es (P.L.); noailles24@gmail.com (A.N.); 2 Department of Optics, Pharmacology and Anatomy, University of Alicante, 03690 Alicante, Spain; E-Mails: angulo@ua.es (A.A.); vmaneu@ua.es (V.M.)

**Keywords:** retina, apoptosis, oxidative stress, *Crocus sativus*, safranal, tauroursodeoxycholic acid, TUDCA, P23H

## Abstract

All retinal disorders, regardless of their aetiology, involve the activation of oxidative stress and apoptosis pathways. The administration of neuroprotective factors is crucial in all phases of the pathology, even when vision has been completely lost. The retina is one of the most susceptible tissues to reactive oxygen species damage. On the other hand, proper development and functioning of the retina requires a precise balance between the processes of proliferation, differentiation and programmed cell death. The life-or-death decision seems to be the result of a complex balance between pro- and anti-apoptotic signals. It has been recently shown the efficacy of natural products to slow retinal degenerative process through different pathways. In this review, we assess the neuroprotective effect of two compounds used in the ancient pharmacopoeia. On one hand, it has been demonstrated that administration of the saffron constituent safranal to P23H rats, an animal model of retinitis pigmentosa, preserves photoreceptor morphology and number, the capillary network and the visual response. On the other hand, it has been shown that systemic administration of tauroursodeoxycholic acid (TUDCA), the major component of bear bile, to P23H rats preserves cone and rod structure and function, together with their contact with postsynaptic neurons. The neuroprotective effects of safranal and TUDCA make these compounds potentially useful for therapeutic applications in retinal degenerative diseases.

## 1. Introduction

The retina is the light-sensitive tissue that lines the inner surface of the eye and is in charge of the first steps of visual processing. The structural and functional complexity of the retina makes this tissue vulnerable to alterations from any sort of pathological injury. At the cellular and molecular level, the response of the retina to injury is similar in all retinal neurodegenerative diseases, and results in a set of cell signals that lead to well-established and similar morphological and functional impairments. Regardless of the aetiology of the damage, all retinal disorders involve the activation of oxidative stress and apoptosis pathways [1].

The retina is one of the most susceptible tissues to reactive oxygen species (ROS) damage. Photoreceptor cells are continuously exposed to light, and are one of the highest consumers of oxygen in the central nervous system (CNS). In this context, ellipsoids of photoreceptors exhibit a high density of mitochondria [2] that provide energy for phototransduction and the maintenance of Ca^2+^ homeostasis [3]. Moreover, experimental evidences show the presence of aerobic metabolism in the membranous disks of photoreceptor outer segments [4,5]. The energy required for synaptic vesicular trafficking and regulation of the cytosolic Ca^2+^ levels in the presynaptic terminals of photoreceptors is provided by mitochondria present in both rod spherules and cone pedicles [6]. Dysfunctional mitochondria cause an energy deficit, leading to an increase of ROS levels and an abnormal elevation of cytosolic Ca^2+^ [7,8]. It is widely accepted that oxidative stress plays a central role in retinal degeneration. Thus, for example, oxidative stress associated with smoking has been considered one of the most important risk factors in the development of age-related macular degeneration (AMD) [9,10]. In retinitis pigmentosa (RP) and glaucoma, the apoptotic stimuli are also exacerbated by oxidative stress [11,12,13]. In diabetic retinopathy (DR), hyperglycemia drives mitochondria to increase their activity [14,15], which results in an overproduction of superoxide from mitochondria [16,17,18].

Proper development and functioning of the retina requires a precise balance between the processes of proliferation, differentiation and programmed cell death. Most defective, unwanted and potentially dangerous cells die by apoptosis, a controlled genetic program for removing cells without damaging the surrounding tissue [19]. The life-or-death decision seems to be the result of a complex balance between pro- and anti-apoptotic signals [20,21,22]. The progressive loss of vision via photoreceptor cell death is a shared trait of all inherited retinal diseases [22,23]. The types of cell death within the affected retina are variable, with different or multiple forms of cell death discovered in different models of the same disease [23,24,25]. In human samples and animal models of retinitis pigmentosa, the morphological characteristics of apoptosis have been described [26,27,28], even though more recent evidences suggest that photoreceptor cell death may result primarily from non-apoptotic mechanisms [23,24,25].

Retinitis pigmentosa constitutes a large heterogeneous group of inherited neurodegenerative retinal disorders that cause a progressive loss of retinal function and represent a major cause of blindness. More than 100 different mutations in the rhodopsin-encoding gene (RHO) are associated with RP, together accounting for 30% to 40% of autosomal dominant RP (adRP) cases [29]. The P23H mutation in RHO is the most prevalent cause of adRP [30], which alone accounts for approximately 12% of adRP cases in the United States [31]. The majority of RP-causing mutations in the RHO gene, including P23H, cause misfolding and retention of rhodopsin in the endoplasmic reticulum [32]. These studies also suggest that the mechanism of RP involves a cellular stress response [33], the final common pathway being programmed during photoreceptor cell death [34]. Transgenic P23H rats mimic the clinical findings reported for human patients with P23H RP [35,36]. These animals develop a progressive rod dysfunction, albeit initially exhibiting a normal cone function. The loss of photoreceptors is accompanied by degenerative changes in the inner retina [37], including a substantial degeneration of retinal ganglion cells [38,39,40]. P23H line 3 rats retain vision for relatively long periods of their lives, similarly to findings in P23H humans, who exhibit significantly better visual acuity and greater ERG amplitudes than patients harboring other RP mutations [35,36].

Neuroprotective treatments provide therapeutic strategies independent of the aetiology of the degeneration. The aim of neuroprotective mechanisms is to provide an adequate environment in which to prolong the viability of retinal cells through their effects on a number of biochemical pathways. This can be achieved by either delivering neurotrophic growth factors to retinal tissues, inhibiting pro-apoptotic pathways or implementing viability factors. In this context, traditional medicine provides frontline pharmacotherapy for many millions of people worldwide. In this review, we will discuss the efficacy of two natural compounds, safranal and TUDCA, to slow retinal degenerative process through different pathways. The administration of neuroprotective factors, which slow retinal degeneration, may be crucial in all phases of the pathology, even when vision has been completely lost.

## 2. Results and Discussion

Human retinal degenerative diseases are currently incurable and retinal degeneration, once initiated, is irreversible. The therapies applied at present in the treatment of retinal dystrophies are focused on delaying the onset or progression of degeneration, but no therapies are available to replace lost retinal cells or restore accurate vision. Currently, the potential therapeutic approaches aimed at finding a cure for blinding diseases focus on two main lines of action. First is the use of preventive strategies that attempt to counteract the underlying disease mechanisms, either by manipulating cellular pathways through the use of pharmacological compounds or genetic modification by gene silencing and/or gene replacement. The second approach is not concerned as much with the causes of the diseases as it is with ways to prevent cell death, such as the administration of anti-apoptotic, anti-inflammatory and neurotrophic compounds. In this context, the administration of antioxidants (alone or in cocktails), anti-apoptotics, anti-inflammatories, neurotrophic factors or viability factors may slow the neurodegeneration of the retina by delaying retinal cell death [41,42,43,44].

### 2.1. Saffron Constituents Prevent Vision Loss and Retinal Degeneration

The pistil of *Crocus sativus*, commonly known as saffron, has been commonly used in traditional medicine as an anodyne, sedative and emmenagogue. In traditional Islamic medicine, they are attributed a wide range of activities: such as oxytocic, anti-carcinogenic, exhilarant, anti-depressant, and anti-asthma effects [45,46]. More recently, the active constituents of saffron (crocetin, crocin and safranal) have been described as powerful carotenoid antioxidants with protective and therapeutic properties [47,48,49].

Extracts from saffron have been used to delay retinal degeneration in early stages of age-related macular degeneration [50] and in a rodent model of light-induced retinal damage [51]. In albino rats fed on saffron supplements, the effects of continuous bright light exposure were significantly diminished, and the morphology and function of the retina were maintained [51,52,53]. Additionally, in clinical trials involving human patients with early AMD, 20 mg per day of saffron supplementation for 90 days significantly improved some parameters of the macular photopic flash electroretinogram, such as amplitude and modulation threshold [50].

Crocetin is one of the major active compounds in saffron [54]. Crocetin has been reported to prevent retinal degeneration induced by oxidative and endoplasmic reticulum stresses via the inhibition of caspase 3 and 9 activities in the RGC-5 retinal ganglion cell line *in vitro* and in a mouse model of light-induced retinal degeneration (LIRD) *in vivo* [55]. Moreover, crocetin inhibited retinal ischemic damage in mice, preventing the apoptotic death of ganglion cells and the reduction of the inner nuclear layer (INL) by decreasing the activation of mitogen-activated protein kinases (p38, JNK) and redox-sensitive transcription factors (NF-κB and c-Jun), while maintaining the functional activity of the retina [56]. Crocetin also prevented NMDA-induced murine retinal damage by inhibiting both caspases 3 and 7 activation and the increased expression of cleaved caspase 3 in the ganglion cell layer (GCL) and INL [57].

The carotenoid crocin is one of the constituents of saffron stigmas [54]. Treatment with crocin protected retinal photoreceptors against light-induced cell death in primary cell cultures from primate and bovine retinas [58]. Cell death was significantly attenuated in cell cultures pretreated with various concentrations of crocin. On the other hand, crocin analogs were found to increase the blood flow in the retina and choroid and to facilitate retinal function recovery [59]. In rat models of retinal ischemia/reperfusion, crocin prevented injury-induced apoptosis of RGCs by activating the PI3K/AKT signaling pathway [60].

**Figure 1:**
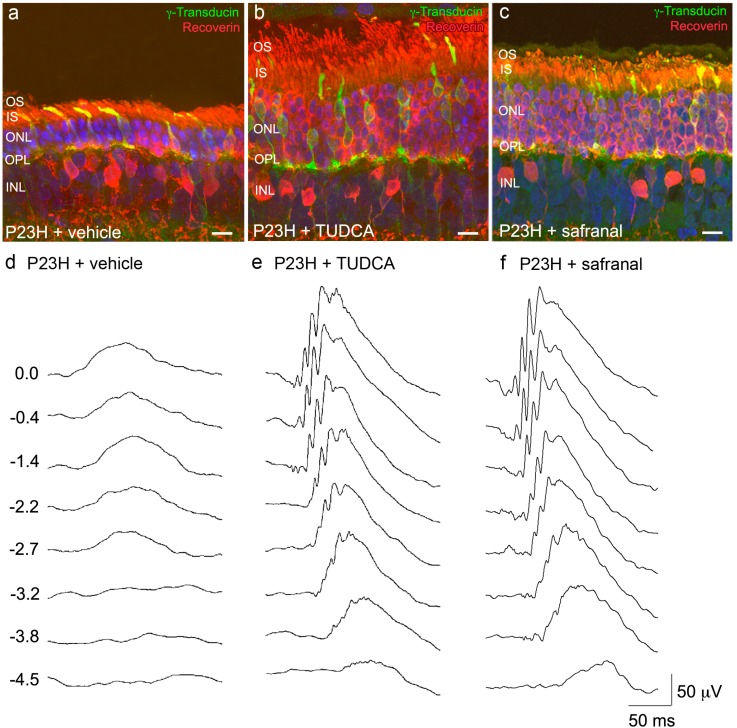

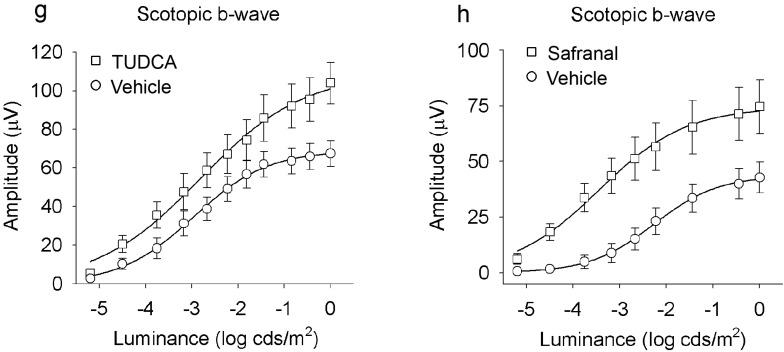
Neuroprotective effects of TUDCA and safranal on the morphological and functional changes associated to retinal degeneration. (**a**–**c**) Immunolabeling of retinal vertical sections for γ-transducin (cones, green) and recoverin (rods, cones, and two bipolar cell subtypes, red) in P120 P23H rats treated with vehicle (**a**) TUDCA (**b**) or safranal (**c**). Nuclei stained with TO-PRO 3 (blue). Images were collected from the central area of the retina, close to the optic nerve; (**d**–**f**) Representative scotopic full-field ERG waveforms from P120 P23H rats treated with vehicle (**d**) TUDCA (**e**) or safranal (**f**). Units on the left indicate input flash intensities in log cd·s/m^2^. Note that ERG amplitudes in the P23H rat treated with TUDCA of safranal are higher than those recorded in the vehicle-treated animal; (**g**–**h**) Stimulus intensity curves for mixed scotopic b-waves from rats administered with TUDCA (**g**, squares), safranal (**h**, squares) or vehicle (**g**–**h**, circles). OS: outer segments, IS: inner segments, ONL: outer nuclear layer, OPL: outer plexiform layer, INL: inner nuclear layer. Scale bars: 10 μm.

Safranal has been seen to prevent both the decrease of antioxidant enzyme activities and lipid peroxidation occurring with age in rat livers [61]. In ischemic rats, safranal also exerted a protective action against oxidative damage in skeletal muscle [62] and cerebral tissues [63]. Administration of the saffron constituent safranal to P23H rats, an animal model of retinitis pigmentosa, preserved photoreceptor morphology and number, and the visual response (Figure 1) [42]. The analysis of the capillary network revealed that safranal is also able to prevent the loss of retinal vessels that occurs in the P23H rat retina [42], evidencing more extensive capillary networks and better-preserved capillary loops, as compared to untreated animals. These evidences suggest that the neuroprotective action of safranal extends not only to photoreceptor cells, but also to other retinal cells.

### 2.2. Bear Bile Constituents Prevent Vision Loss and Retinal Degeneration

Bear bile has been used in traditional Chinese medicine for over 3000 years to treat visual disorders [64]. Tauroursodeoxycholic acid (TUDCA), the major component of bear bile, has been shown to exhibit antiapoptotic properties in neurodegenerative diseases, including those affecting the retina. Systemic administration of TUDCA has been demonstrated to slow retinal degeneration in both the rd10 autosomal recessive RP mouse model [64,65,66,67,68] and in a LIRD mouse model [67]. In these two retinal degeneration models, TUDCA-treated animals were shown to maintain better visual function, thicker outer nuclear layer (ONL) and better preservation of outer segments than untreated animals. TUDCA also prevented retinal degeneration in the P23H autosomal dominant RP rat model (Figure 1) [41]. P23H treated rats showed higher a- and b-wave amplitudes under both photopic and scotopic conditions than untreated rats. Moreover, TUDCA decreased photoreceptor apoptosis and maintained synaptic connectivity among retinal cells [41]. Furthermore, P23H rats treated with TUDCA exhibited greater labeling for cytochrome c oxidase subunit IV in photoreceptor cell mitochondria, which is closely related to improved physiological activity and oxidative metabolism [6].

In addition to its anti-apoptotic properties, TUDCA has also been shown to exert anti-inflammatory, antioxidant and chaperone activities. In this context, TUDCA suppressed the formation of laser-induced choroidal neovascularization (CNV) in rats by decreasing the number and size of CNV lesions, probably due to its anti-inflammatory properties, which diminished vascular endothelial growth factor (VEGF) levels in the retina after the laser treatment [69]. Additionally, systemic administration of TUDCA preserved photoreceptors after retinal detachment in rats, preventing the reduction in ONL thickness, and this was accompanied by decreased oxidative stress and inhibition of the increase in caspase 3 and 9 activity [70]. TUDCA also protected retinal neural cell cultures from high glucose-induced death by decreasing mitochondrial-nuclear translocation of the apoptosis inducing factor (AIF). This inhibition of the release of AIF from the mitochondria was probably due to the antioxidant properties of TUDCA, as corroborated by the marked decrease in oxidative stress biomarkers with TUDCA treatment [71]. These findings may have relevance in the treatment of DR. Furthermore, systemic injection of TUDCA diminished endoplasmic reticulum stress, prevented apoptosis and reduced cone degeneration in the retina of a mouse model of Leber congenital amaurosis [72].

### 2.3. TUDCA Reduces Microglial Cell Activation in Degenerative Retinas

Microglial cells in the retina act as sensors of disarrangement in their microenvironment. Their balanced activities play a key role in the survival of neurons [73,74]. Activation of the microglia has been demonstrated in association with several neurodegenerative diseases, such as Alzheimer’s and Parkinson’s diseases, amyotrophic lateral sclerosis, and multiple sclerosis, although it remains unclear whether microglial activation is a cause or a consequence of neuronal damage [75,76,77,78,79].

**Figure 2:**
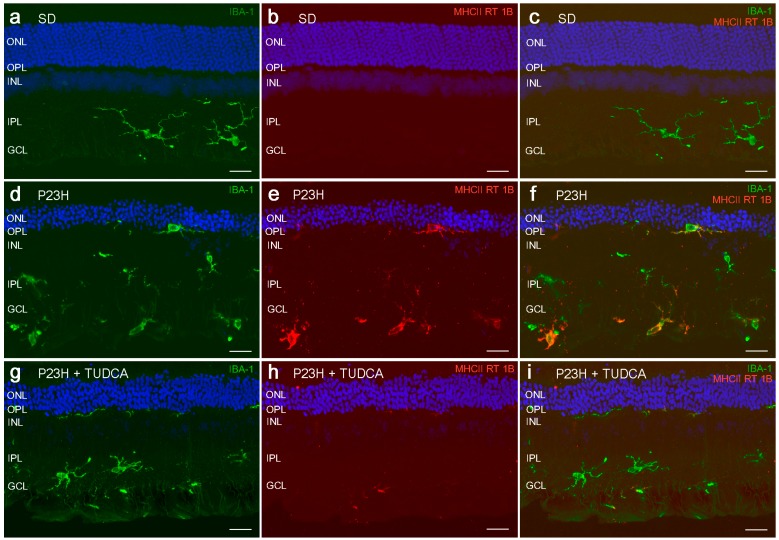
Activation of microglial cells. Vertical sections of retinas from a SD (**a**–**c**), untreated P23H (**d**–**f**) and TUDCA-treated P23H (**g**–**i**) rat at P120 stained for Iba1 (green; **a**, **d**, **g**) MHC-II RT 1B (red; **b**, **e**, **h**) or both (**c**, **f**, **i**). Nuclei stained with TO-PRO 3 (blue). All images were collected from the central area of the retina, close to the optic nerve. Note that microglia density in P23H rats treated with TUDCA is similar to the one observed in the SD rats and smaller than that shown in untreated P23H rats. The relative number of MHC-II-positive cells in TUDCA-treated P23H rats is also low. ONL: outer nuclear layer, OPL: outer plexiform layer, INL: inner nuclear layer, IPL: inner plexiform layer, GCL: ganglion cell layer. Scale bars: 20 μm.

Changes in microglial cells number, activation and distribution have been reported in different forms of disease or retinal damage, like glaucoma [80,81], age-related macular degeneration [82,83], light damage [84,85] and retinitis pigmentosa [86]. In P23H rat retinas, microglia density increased in the GCL, IPL (inner plexiform layer) and OPL (outer plexiform layer), microglial cells appears in the subretinal space (SS), and a great deal of microglial cells were labeled with anti-MHC-II, a marker of microglia activation (Figure 2). In the mouse model of retinitis pigmentosa rd10, it has been demonstrated high levels of pro-inflammatory cytokines and chemokines and early microglia activation [87,88]. Previous studies have also demonstrated increased density of macrophages after microglia activation [89].

Systemic administration of TUDCA reduced the number and activation of microglial cells in P23H rats. Moreover, in TUDCA-treated rat retinas microglia was mainly distributed in more internal retinal layers, GCL and IPL, and they are scarce in the OPL and missing in the SS, similar to that found in normal rat retinas (Figure 2). Attenuation of microglial activation using TUDCA has been also demonstrated in experimental models of neuroinflammation, in which it has been reported that TUDCA reduces *in vitro* microglial migration and the expression of chemoattractants required for microglial migration [90]. The effects of TUDCA on retinal microglial cells could be also attributed to an effect of TUDCA on microglial cells behavior, presumably interfering in the respiratory burst of the microglia, which is a critical step in its activation [71,91].

### 2.4. Neuroprotective Mechanisms

**Figure 3:**
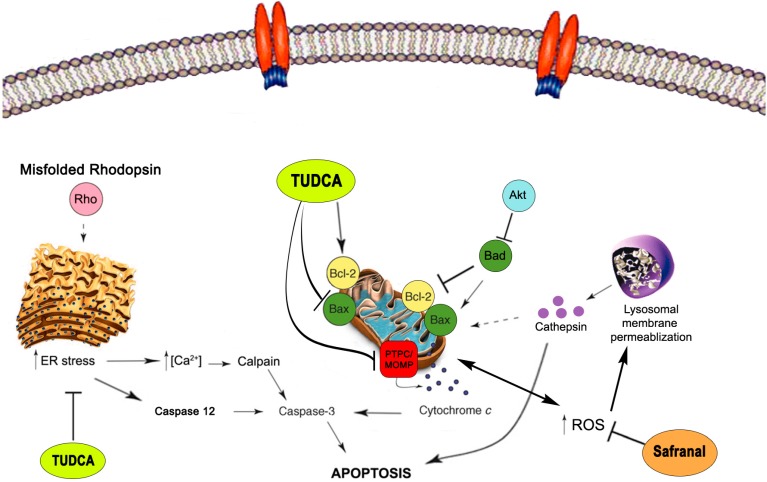
Apoptotic pathways in the retina. Schematic representation of the most relevant pathways involved in programmed cell death (PCD) in the P23H rat retina and likely targets for safranal and TUDCA. Most retinal cells die as a result of caspase-dependent pathways, although caspase-independent pathways involving calpains and/or cathepsins are also present. TUDCA activity may be exerted by decreasing ER stress, stabilizing the outer mitochondrial membrane and blocking calpain-driven apoptosis. Among the major causes of stress and cell death in the retina is the accumulation of reactive oxygen species (ROS) associated with pathological conditions and damage to both mitochondria and lysosomes. Safranal could be decreasing oxidative stress due to ROS elevation, thereby ameliorating cell death. Akt/PKB: Protein kinase B; Bad: Bcl-2-associated agonist of cell death; Bax: Bcl-2-associated X protein; Bcl-2: apoptosis regulator Bcl-2 (B-cell lymphoma-2); ER: endoplasmic reticulum; MOMP: mitochondrial outer membrane permeabilization; PTPC: permeability transition pore complex; ROS: reactive oxygen species.

In RP, the high diversity of genes and mutations involved leads to the activation of a variety of apoptotic and non-apoptotic pathways [23,24,28]. In rodent models of RP, endoplasmic reticulum (ER) stress triggers an increase in cytosolic Ca^2+^ levels with ensuing up-regulation of caspase 12 [92], which in turn activates caspase 3. In the P23H rat retina, rod photoreceptor cells have been shown to die due to apoptosis triggered by ER stress, mainly through caspase 12 [93], although activation of alternative pathways could play a significant role in this process [23,24,25]. The accumulation of misfolded proteins and increased cytosolic Ca^2+^ activates additional pro-apoptotic factors that reinforce each other during the apoptosis process (Figure 3) [94]. It has been shown that, besides the activation of caspase 12, ER stress and the subsequent Ca^2+^ release can activate calcium-dependent cysteine proteases known as calpains [95,96]. These enzymes are present in the cytosol, and together with caspase 12 [97] and other pro-apoptotic proteins [96], may amplify the death signal. The activation of calpains has been related to various retinal diseases, and is considered to be one of the most important caspase-independent apoptotic pathways in photoreceptor cell death associated with RP [11,28,98,99]. In this scenario, mitochondria play an important role in apoptosis, due to their rich content in pro-apoptotic proteins (Figure 3) [100]. Bcl-2 is the best-characterized protein family involved in the progression of apoptosis in photoreceptor cells. A decrease in the ratio of Bcl-XL to Bax (Bcl-2-associated X protein) has been evidenced in RP animal models carrying mutations in the rhodopsin gene, thus indicating the implication of mitochondria in the progress of apoptosis [93,101]. In this context, preserving the integrity of the mitochondrial membrane by preventing the formation of mitochondrial outer membrane pores (MOMP) and the modulation of existing mitochondrial channels, such as the mitochondrial permeability transition pore complex, could be good anti-apoptotic strategies to protect cells from death [102,103].

In P23H rats, it has also been demonstrated that mTOR/Akt and autophagy signaling, along with the expression of Bcl-2 family proteins, is altered during adRP progression, and this correlated with Ca^2+^ changes promoted by calpain and caspase 12 activation. ROS accumulation may also induce mitochondrial and lysosomal membrane permeabilization, releasing pro-apoptotic proteins [23,104,105,106,107].

The exact mechanisms by which safranal and TUDCA exert neuroprotection remain unclear. Our hypotheses concerning the possible pathways in which these compounds could act in order to counterbalance apoptosis in the P23H retina are schematized in Figure 3.

### 2.5. Neuroprotective Mechanisms of Safranal

Saffron and its constituents, such as safranal, have been shown to exert cytoprotective effects on a wide spectrum of tissues, including nervous tissue, in a dose-dependent manner [63]. The best described mechanism by which saffron acts against cell damage is its antioxidant activity (Figure 3), relying on its ability to scavenge free radicals [108]. Also, this compound promotes an increase of the natural antioxidant defenses of the cell [61], together with its capacity to protect DNA and RNA from harmful chemical reactions by forming ligand-polynucleotide complexes [109,110].

The retina is one of the tissues with the highest oxygen consumption in the body of mammals. Retinal degeneration is accompanied by an increase in ROS and an abnormal rise in cytosolic Ca^2+^, which are able to permeabilize the mitochondrial [102] and lysosomal membranes, thereby amplifying the pro-apoptotic events [105,111]. In this scenario, safranal could decrease the activation of different apoptotic pathways that may be stimulated by the increase in ROS (Figure 3). In addition to this high level of antioxidant activity, safranal has also been shown to be capable of downregulating Bax and caspase 3 expression in muscle cells of rats with myocardial ischemia-reperfusion injury [112], suggesting an additional antiapoptotic activity. All these results have contributed to initiate clinical trials aimed at testing the activity of saffron in patients with age-related macular degeneration [49] based on the success of previous studies in animal models [50,51].

### 2.6. Neuroprotective Mechanisms of TUDCA

The activity of TUDCA has been widely investigated in several models of neurodegenerative diseases. Previous works have demonstrated that TUDCA is able to modulate apoptosis by regulating different pro-apoptotic pathways [113,114]. This may be related to the neurotoxicity caused by accumulation of amyloid beta β in cells, as it occurs in Alzheimer disease [115,116]. It has been observed that TUDCA inhibits β-induced mitochondrial permeabilization and cytocrome c release in primary rat neurons and astrocyte cultures [117] and is able to stabilize the mitochondrial membrane, thereby preventing Bax translocation and MOMP formation in isolated mitochondria [118]. In addition, TUDCA has been described as a chemical chaperone capable of reducing the ER stress caused by misfolded proteins in diabetic retinopathy models [119,120] and in cases of retinal degeneration involving ER stress [72,121]. TUDCA protects against ER stress presumably by modulating intracellular Ca^2+^ levels and thereby inhibiting calpains and caspase 12 activation (Figure 3) [119,122].

All these features make TUDCA a good therapeutic agent, due to its effectiveness not only in delaying photoreceptor cell loss, but also in slowing the degeneration of inner retinal cells. Therefore, TUDCA might be useful in combination with antioxidants and anti-inflammatory agents, together with therapies based on gene therapy or the transplantation of new photoreceptors, to further ensure the success of the latter. Researchers are also currently focusing on different strategies to deliver this compound to the retina. Systemic administration of TUDCA requires high concentrations to be effective against retinal tissue neurodegeneration [41,65,66,67,68,69,70,71,72]. Although TUDCA is well tolerated at high doses, new delivery mechanisms are being explored in order to achieve effective concentrations of this drug in the retina without extensive spreading to other tissues. In this sense, administration by intravitreal injection of TUDCA-loaded microspheres seems to provide all the advantages of the treatment, while using concentrations of this compound lower than those used in systemic administration [123].

## 3. Conclusions

The present review shows the capacity of the natural compounds safranal and TUDCA to delay retinal degeneration. Both safranal and TUDCA were able to reduce rod and cone photoreceptor loss, as well as to protect their electroretinographic response in the P23H adRP rat model. The synaptic contacts between photoreceptors and their postsynaptic cells were also more numerous and better preserved as compared to untreated animals in all experiments. The treatments were also able to diminish the degree of secondary remodeling that takes place in the retina after photoreceptor cell death, improving the condition of cells postsynaptic to photoreceptors. Moreover, we documented that TUDCA reduces the number and activation of microglial cells in the P23H model of RP. The neuroprotective effects of safranal and TUDCA make these natural compounds potentially useful for delaying retinal degeneration in retinal pathologies.
